# Optimization of antibiotic use in the intensive care unit: how we do it

**DOI:** 10.62675/2965-2774.20240017-en

**Published:** 2024-10-11

**Authors:** Patrícia Moniz, João Fustiga, Marta Maio Herculano, Pedro Póvoa

**Affiliations:** 1 Centro Hospitalar de Lisboa Ocidental Department of Intensive Care Medicine Lisbon Portugal Department of Intensive Care Medicine, Centro Hospitalar de Lisboa Ocidental - Lisbon, Portugal.; 2 Universidade Nova de Lisboa NOVA Medical School Lisbon Portugal NOVA Medical School, Universidade Nova de Lisboa - Lisbon, Portugal

## INTRODUCTION

Antibiotic stewardship program (ASP) implementation in the intensive care unit (ICU) remains a challenge. Severe infections and the increasing prevalence of multidrug-resistant pathogens indicate the need for judicious antibiotic use. However, the global health threat of antibiotic resistance, with up to 1.2 million attributable deaths worldwide, encourages intensivists to reduce antibiotic consumption.^(1)^ Moreover, pharmacokinetic (PK) and pharmacodynamic (PD) derangements in critical illness are associated with over- and underdosing.^(2)^

All ASPs should have the aim of reducing antibiotic consumption and consequently preventing adverse reactions and reducing resistance. Healthcare-associated infections and antimicrobial resistance are associated with high healthcare costs and mortality, with a significant prevalence worldwide and even higher incidences in developing countries.^(3)^ In addition to restrictive clinical interventions, structural modifications in ICU dynamics, such as antibiotic consumption surveillance systems, staff participation, ICU leadership interventions and collaboration with other health care professionals, such as pharmacists, microbiologists and infection control units, are crucial.^(4)^ Although ASPs are encouraged and their importance is unquestionable, their implementation in ICUs requires multiple measures and remains suboptimal. We present this viewpoint commentary focusing on relevant ASP measures and the respective obstacles we may encounter with antibiotic therapy (ABT) optimization.

## WHEN AND HOW TO START ANTIBIOTICS

Antibiotic prescription should be timely, appropriate, and adequate. An evaluation of the likelihood of infection is needed to guide antibiotic prescriptions while minimizing their harm, especially in immunosuppressed or critically ill patients.^(1)^ The Surviving Sepsis Campaign (SSC) guidelines recently focused on the severity of illness and recommended antibiotic therapy in the first hour for septic shock.^(2)^ When the suspicion of infection is uncertain and the patient does not present new-onset organ dysfunction secondary to probable underlying bacterial infection, we prioritize the microbiological approach with antibiotic prescription initiation depending on pathogen identification.

Appropriate empiric ABT should be based on proven *in vitro* activity against the causative pathogens.^(5)^ Adequate therapy emphasizes the optimal dosage and administration routes for penetration at targeted infection sites.^(2)^ Patients with septic shock require prompt empirical therapy and present higher mortality with inadequate regimens.^(6)^ Moreover, an overly broad antibiotic spectrum has been associated with higher mortality than inappropriate therapy.^(7)^

Another pillar of ASP is microbiological sampling,^(1,2)^ with at least two sets of blood cultures obtained in addition to other samples, according to the suspected foci of infection. Ideally, all cultures should be collected before antibiotic initiation. If such timing is not possible, the need for sample collection should not delay ABT administration. Source control should be sought whenever needed.^(2)^

Empiric methicillin-resistant *Staphylococcus aureus* (MRSA) coverage, regardless of infection severity, is often unnecessary.^(1)^ The current recommendations contribute to excess anti-MRSA therapy, potentially leading to morbidity and mortality.^(8,9)^ We only institute MRSA coverage in patients with known risk factors, compatible sources of infection and evidence of MRSA nasal colonization. Contradictory data exist regarding the use of carbapenem-sparing agents for extended-spectrum beta-lactamase infections.^(8,9)^ Antibiotic stewardship programs have the aim of decreasing carbapenem use, emphasizing the need for evidence-based practices.

## THE IMPORTANCE OF PHARMACOKINETICS AND PHARMACODYNAMICS IN CRITICALLY ILL

Pharmacokinetics is markedly altered in critically ill patients. The SSC guidelines recommend optimizing antibiotic dosing strategies on the basis of drug properties and PK/PD principles. ^(2)^

Altered PK in critically ill patients, such as an increased volume of distribution (Vd) and augmented renal clearance, poses for the risks of suboptimal dosing and therapeutic failure. In the initial approach to sepsis and septic shock, capillary leakage and aggressive intravenous resuscitation promote Vd expansion.^(10)^ Hydrophilic antibiotics, such as aminoglycosides, β-lactams, and glycopeptides with low Vd, require high loading doses to reach therapeutic concentrations. Furthermore, in septic shock, absorption issues should promote the implementation of intravenous drug administration.^(11)^ Therefore, we advocate for higher loading doses, even amidst renal failure, for at least 24 to 48 hours, when capillary leakage and fluid resuscitation are more pronounced.

Pharmacodynamics reflect the parameters of antibiotic activity and rely on the minimal inhibitory concentration (MIC). If the MIC increases, PK exposure should also increase to guarantee an optimal PK/PD index.^(11)^ Our unit takes MIC values into consideration when de-escalation is possible and sensitivity testing is available by discussing these issues with the microbiology lab.

The recommendations in recent studies have suggested the use of prolonged rather than intermittent administration of β-lactams because of their time-dependent bactericidal activity.^(12,13)^ Our practice consists of prolonged or continuous β-lactam infusions, according to the drug stability, after an initial loading dose.

Therapeutic drug monitoring, which was initially implemented due to fear of toxicity, has been advocated because of the risks of suboptimal dosing.^(12)^ In addition to the use of aminoglycosides and vancomycin, we recently adopted therapeutic drug monitoring for β-lactams in the acute phase of septic shock or when toxicity is a concern. However, these timely procedures do not provide immediate results, hindering decision-making.

## WHEN AND HOW TO ADJUST ANTIBIOTICS

The possibility of antibiotic de-escalation (ADE) is assessed daily in our ICU. The ADE does not seem to impact mortality,^(14)^ and it has been proven to be safe. Its implementation seems reasonable because reducing antibiotic exposure should help prevent antibiotic resistance.

A variety of measures, such as narrowing of the antibiotic spectrum, avoidance of broad-spectrum antibiotics, and switching from combination therapy to monotherapy, constitute examples of ADE.^(1)^ If the patient demonstrates clinical improvement and the underlying pathogens and susceptibilities are identified, ADE should be encouraged. Moreover, even if the pathogens remain unknown, de-escalation remains appropriate in clinically improved patients.^(2)^

Antibiotic de-escalation has been shown to be associated with increased ABT duration. However, this relationship may be related to the heterogeneous patient groups and psychological factors affecting physicians advocating for longer narrow-spectrum regimens for "safety reasons". In the first randomized controlled trial (RCT) testing de-escalation, Leone et al. showed that ADE measures seemed safe and decreased combination therapy and antipseudomonal agent exposure.^(14)^

## DEFINING THE DURATION AND TIME TO STOP

Longer antibiotic courses of overly broad-spectrum therapies do not improve patient outcomes and lead to an increased risk of secondary healthcare-associated infections and higher costs.^(15)^ Moreover, short antibiotic courses have been shown to be safe and effective. Although shorter courses are recommended, compliance is low, and the duration of ABT remains excessive.^(4)^

Intensivists are well aware of the importance of antibiotic initiation. However, the possibility of antibiotic discontinuation should also be prioritized, especially in the absence of infection. The decision to stop strongly depends on the correct and timely collection of microbiological samples. In patients who have clinically improved and whose quantitative cultures performed before antibiotic initiation are negative, antibiotics should be stopped.^(1)^ In patients with microbiologically confirmed infection, the duration of ABT depends on the clinical course, the site of infection and the pathogen. Regardless of the site of infection, adequate source control, whether with a medical or surgical approach, must be guaranteed.^(2)^

Biomarker kinetics have also been studied as a strategy to guide clinicians in ABT decision-making. Randomized controlled trials and meta-analyses have assessed strategies using host-response biomarkers, such as procalcitonin and C-reactive protein (CRP), to predict and diagnose sepsis, assess sepsis response and guide ABTs and revealed strong evidence of their safety and the possibility of shorter antibiotic durations. Serial, instead of single, determinations of these biomarkers are more informative and should always be integrated in the clinical context. This strategy of use depends on a thorough understanding of their biology, strengths, and limitations. However, questions remain regarding the efficacy and costs of this biomarker-guided strategy in ASP.^(4,16)^

We support shorter durations of ABT in patients who have clinically improved and demonstrate adequate source control and favorable biomarker evolution on the basis of CRP kinetics. In patients with negative but adequate microbiological samples collected prior to ABT administration, we favor ABT suspension.

**Figure 1:**
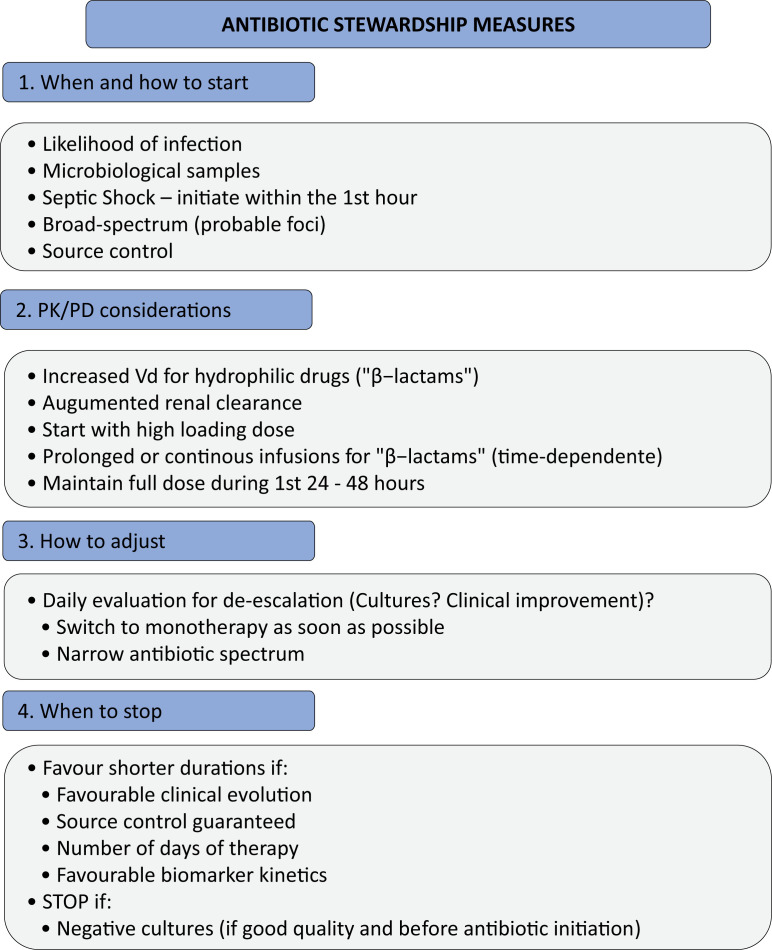
Proposed strategies to guide antibiotic stewardship implementation.

Figure 1 shows our proposed ASP strategy with the previously discussed measures.

PK/PD - Pharmacokinetic/pharmacodynamic; Vd - volume od distribution.

## CONCLUSION

The authors present plausible strategies for adequate antibiotic stewardship program measures by focusing on aspects of antibiotic initiation, pharmacokinetic/pharmacodynamic characteristics influencing dosing strategies, antibiotic de-escalation, and duration determination.

The unquestionable importance of antibiotic initiation regarding timing, appropriateness, and adequacy unsurprisingly leads to high antibiotic pressure in the intensive care unit. As intensivists, we share the responsibility of judicious antibiotic use and should lead the way with regard to antibiotic stewardship program implementation. Nevertheless, antibiotic stewardship programs should integrate multidisciplinary discussions for antibiotic stewardship optimization.
