# Supplementary material for: Optimization of antibiotic use in the intensive care unit: how we do it
Source: Crit Care Sci. 2024 Oct 11;36:e20240017en. doi: 10.62675/2965-2774.20240017-en (PMC11554294; doi:10.62675/2965-2774.20240017-en)
Supplement: Translation

# Translation: Otimização do uso de antibióticos na unidade de terapia intensiva: como fazemos

**DOI:** 10.62675/2965-2774.20240017-pt

**Authors:** Patrícia Moniz, João Fustiga, Marta Maio Herculano, Pedro Póvoa

**Affiliations:** 1 Lisboa Portugal Departamento de Medicina Intensiva, Centro Hospitalar de Lisboa Ocidental - Lisboa, Portugal.; 2 Lisboa Portugal NOVA Medical School, Universidade Nova de Lisboa - Lisboa, Portugal

## INTRODUÇÃO

A implementação do *Antimicrobial Stewardship Program* (ASP) na unidade de terapia intensiva (UTI) continua um desafio. Infecções graves e a crescente prevalência de patógenos multirresistentes indicam a necessidade do uso criterioso de antibióticos. No entanto, a ameaça global à saúde pela resistência aos antibióticos, com até 1,2 milhão de mortes atribuíveis em todo o mundo, incentiva os intensivistas a reduzirem o uso de antibióticos.^(1)^ Além disso, os distúrbios farmacocinéticos (PK) e farmacodinâmicos (PD) em doenças graves estão associados à superdosagem e à subdosagem.^(2)^

Todos os ASPs devem ter como objetivo reduzir o uso de antibióticos e, consequentemente, prevenir reações adversas e reduzir a resistência. As infecções associadas à assistência à saúde e à resistência antimicrobiana estão associadas a altos custos de assistência à saúde e à mortalidade, com prevalência significativa em todo o mundo e incidência ainda maior em países em desenvolvimento.^(3)^ Além de intervenções clínicas restritivas, são cruciais modificações estruturais na dinâmica da UTI, como sistemas de vigilância de uso de antimicrobianos, participação da equipe, intervenções da liderança da UTI e colaboração com outros profissionais de saúde, como farmacêuticos, microbiologistas e unidades de controle de infecções.^(4)^ Embora os ASPs sejam incentivados e sua importância seja inquestionável, sua implementação nas UTIs exige várias medidas e continua abaixo do ideal. Apresentamos este comentário de ponto de vista com foco em medidas de ASPs relevantes e os respectivos obstáculos que podemos encontrar com a otimização da antibioticoterapia (ABT).

## QUANDO E COMO INICIAR OS ANTIBIÓTICOS

A prescrição de antibióticos deve ser oportuna, apropriada e adequada. É necessária uma avaliação da probabilidade de infecção para orientar as prescrições de antibióticos e, ao mesmo tempo, minimizar seus danos, especialmente em pacientes imunossuprimidos ou gravemente enfermos.^(1)^ As diretrizes da *Surviving Sepsis Campaign* (SSC) recentemente se concentraram na gravidade da doença e recomendaram a ABT na primeira hora do choque séptico.^(2)^ Quando a suspeita de infecção é incerta, e o paciente não apresenta disfunção orgânica de início recente secundária a uma provável infecção bacteriana subjacente, priorizamos a abordagem microbiológica com o início da prescrição de antibióticos, dependendo da identificação do patógeno.

A ABT empírica apropriada deve se basear na atividade comprovada *in vitro* contra os patógenos causadores.^(5)^ A terapia adequada enfatiza a dosagem ideal e as vias de administração para penetração nos locais de infecção visados.^(2)^ Pacientes com choque séptico requerem ABT empírica imediata e apresentam maior mortalidade com esquemas inadequados.^(6)^ Além disso, um espectro antibiótico excessivamente amplo foi associado à maior mortalidade do que a ABT inadequada.^(7)^

Outro pilar do ASP é a amostragem microbiológica,^(1,2)^ com pelo menos dois conjuntos de hemoculturas obtidas, além de outras amostras, conforme os focos suspeitos de infecção. Idealmente, todas as culturas devem ser coletadas antes do início do uso de antibióticos. Se esse momento não for possível, a necessidade de coleta de amostras não deve atrasar a administração da ABT. O controle da fonte deve ser buscado sempre que necessário.^(2)^

A cobertura empírica de *Staphylococcus aureus* resistente à meticilina (MRSA), independentemente da gravidade da infecção, é muitas vezes desnecessária.^(1)^ As recomendações atuais contribuem para o excesso de terapia anti-MRSA, o que pode levar à morbidade e à mortalidade.^(8,9)^ Só instituímos a cobertura de MRSA em pacientes com fatores de risco conhecidos, fontes compatíveis de infecção e evidência de colonização nasal por MRSA. Existem dados contraditórios sobre o uso de agentes poupadores de carbapenêmicos para infecções por betalactamases de espectro estendido.^(8,9)^ Os ASPs têm o objetivo de diminuir o uso de carbapenêmicos, enfatizando a necessidade de práticas baseadas em evidências.

## A IMPORTÂNCIA DA FARMACOCINÉTICA E DA FARMACODINÂMICA EM PACIENTES GRAVES

A farmacocinética é marcadamente alterada em pacientes graves. As diretrizes da SSC recomendam a otimização das estratégias de dosagem de antibióticos com base nas propriedades dos medicamentos e nos princípios de PK/PD.^(2)^

A PK alterada em pacientes graves, como o aumento do volume de distribuição (Vd) e o aumento da depuração renal, apresenta riscos de dosagem subótima e falha terapêutica. Na abordagem inicial da sepse e do choque séptico, o extravasamento capilar e a ressuscitação intravenosa agressiva promovem a expansão do Vd.^(10)^ Os antibióticos hidrofílicos, como aminoglicosídeos, betalactâmicos e glicopeptídeos com baixo Vd, exigem altas doses de carga para atingir concentrações terapêuticas. Além disso, no choque séptico, os problemas de absorção devem promover a implementação da administração intravenosa de medicamentos.^(11)^ Portanto, defendemos doses de carga mais altas, mesmo em meio à insuficiência renal, por, pelo menos, 24 a 48 horas, quando o extravasamento capilar e a ressuscitação com fluidos são mais pronunciados.

A PD reflete os parâmetros da atividade antibiótica e depende da concentração inibitória mínima (CIM). Se a CIM aumentar, a exposição à PK também deve aumentar para garantir um índice PK/PD ideal.^(11)^ Nossa UTI leva em consideração os valores de CIM quando existe a possibilidade de redução do uso e quando há testes de sensibilidade, discutindo essas questões com o laboratório de microbiologia.

As recomendações de estudos recentes sugeriram o uso de administração prolongada, em vez de intermitente, de betalactâmicos devido à sua atividade bactericida dependente do tempo.^(12,13)^ Nossa prática consiste em infusões prolongadas ou contínuas de betalactâmicos, de acordo com a estabilidade do medicamento, após uma dose de carga inicial.

O monitoramento terapêutico de medicamentos, que foi inicialmente implementado devido ao temor de toxicidade, tem sido defendido devido aos riscos de dosagem subótima.^(12)^ Além do uso de aminoglicosídeo e vancomicina, recentemente adotamos o monitoramento terapêutico de medicamentos em relação aos betalactâmicos na fase aguda do choque séptico ou quando a toxicidade é uma preocupação. Entretanto, esses procedimentos oportunos não fornecem resultados imediatos, o que dificulta a tomada de decisões.

## QUANDO E COMO AJUSTAR OS ANTIBIÓTICOS

A possibilidade de redução do uso de antibióticos (ADE - *antibiotic de-escalation*) é avaliada diariamente em nossa UTI. A ADE não parece ter impacto na mortalidade,^(14)^ e sua segurança foi comprovada. Sua implementação parece razoável, porque a ADE deve ajudar a prevenir a resistência a antibióticos.

Várias medidas, como estreitar o espectro antibiótico, evitar antibióticos de amplo espectro e mudar de terapia combinada para monoterapia, são exemplos de ADE.^(1)^ Se o paciente demonstrar melhora clínica e os patógenos e suscetibilidades subjacentes forem identificados, deve-se incentivar a ADE. Além disso, mesmo que os patógenos permaneçam desconhecidos, a redução do uso continua apropriada em pacientes com melhora clínica.^(2)^

Foi demonstrado que a ADE está associada ao aumento da duração da ABT. No entanto, essa relação pode estar relacionada a grupos heterogêneos de pacientes e a fatores psicológicos que afetam os médicos que defendem esquemas de espectro restrito mais longos por "razões de segurança". No primeiro estudo controlado randomizado (ECR) que testou a ADE, Leone et al. mostraram que as medidas de ADE pareciam seguras e diminuíram a terapia combinada e a exposição ao agente antipseudomonal.^(14)^

## DEFINIÇÃO DA DURAÇÃO E DO MOMENTO DE INTERROMPER

Protocolos mais longos de antibióticos com terapias de espectro excessivamente amplo não melhoram os desfechos dos pacientes e levam a um aumento do risco de infecções secundárias associadas à assistência à saúde e a custos mais altos.^(15)^ Além disso, foi demonstrado que protocolos curtos de antibióticos são seguros e eficazes. Embora recomendem-se protocolos mais curtos, a adesão é baixa, e a duração da ABT continua excessiva.^(4)^

Os intensivistas estão bem cientes da importância da iniciação da ABT. Entretanto, a possibilidade de interromper o uso de antibióticos também deve ser priorizada, especialmente na ausência de infecção. A decisão de interromper depende muito da coleta correta e oportuna de amostras microbiológicas. Em pacientes que melhoraram clinicamente e cujas culturas quantitativas realizadas antes do início do antibiótico são negativas, os antibióticos devem ser interrompidos.^(1)^ Em pacientes com infecção confirmada microbiologicamente, a duração da ABT depende da evolução clínica, do local da infecção e do patógeno. Independentemente do local da infecção, deve-se garantir o controle adequado da fonte, seja com uma abordagem médica ou cirúrgica.^(2)^

A cinética dos biomarcadores também tem sido estudada como uma estratégia para orientar os clínicos na tomada de decisões sobre a ABT. Metanálises e ECRs avaliaram estratégias que utilizam biomarcadores de resposta do hospedeiro, como procalcitonina e proteína C-reativa (PCR), para prever e diagnosticar a sepse, avaliar a resposta à sepse e orientar ABTs, e revelaram fortes evidências de sua segurança e a possibilidade de menor duração da ABT. Análises seriadas, em vez de individuais, desses biomarcadores são mais informativas e devem sempre ser integradas ao contexto clínico. Essa estratégia de uso depende de uma compreensão completa de sua biologia, pontos fortes e limitações. No entanto, ainda há dúvidas sobre a eficácia e os custos dessa estratégia orientada por biomarcadores no ASP.^(4,16)^

Apoiamos durações mais curtas de ABT em pacientes que melhoraram clinicamente e demonstraram controle adequado da fonte e evolução favorável dos biomarcadores com base na cinética da PCR. Em pacientes com amostras microbiológicas negativas, mas adequadas, coletadas antes da administração da ABT, preferimos a suspensão da ABT.

A figura 1 mostra nossa proposta de estratégia de ASP com as medidas discutidas anteriormente.

**Figura 1:** Estratégias propostas para orientar a implementação do manejo de antimicrobianos.

PK/PD - farmacocinética/farmacodinâmica; Vd - volume de distribuição.

## CONCLUSÃO

Nossos autores apresentam estratégias plausíveis para medidas adequadas do *Antimicrobial Stewardship Program*, concentrando-se em aspectos da iniciação do antibiótico, das características farmacocinéticas/farmacodinâmicas que influenciam as estratégias de dosagem, da redução do uso do antibiótico e da determinação da duração.

A importância inquestionável do início do uso de antibióticos em relação ao momento, à conveniência e à adequação leva, naturalmente, a uma alta pressão antibiótica na unidade de terapia intensiva. Como intensivistas, compartilhamos a responsabilidade do uso criterioso de antibióticos e devemos liderar o caminho com relação à implementação do *Antimicrobial Stewardship Program*. No entanto, os *Antimicrobial Stewardship Programs* devem integrar discussões multidisciplinares para a otimização do manejo de antimicrobianos.
